# C-Reactive Protein Levels in Systemic Lupus Erythematosus Are Modulated by the Interferon Gene Signature and CRP Gene Polymorphism rs1205

**DOI:** 10.3389/fimmu.2020.622326

**Published:** 2021-01-28

**Authors:** Helena Enocsson, Birgitta Gullstrand, Maija-Leena Eloranta, Jonas Wetterö, Dag Leonard, Lars Rönnblom, Anders A. Bengtsson, Christopher Sjöwall

**Affiliations:** ^1^ Department of Biomedical and Clinical Sciences, Division of Inflammation and Infection, Linköping University, Linköping, Sweden; ^2^ Department of Clinical Sciences Lund, Division of Rheumatology, Lund University, Lund, Sweden; ^3^ Department of Medical Sciences, Rheumatology, Uppsala University, Uppsala, Sweden

**Keywords:** C-reactive protein, type I interferons, systemic lupus erythematosus, inflammation, biomarker, pentraxins, interferon, gene variants

## Abstract

**Objectives:**

Patients with systemic lupus erythematosus (SLE) often display modest elevations of C-reactive protein (CRP) despite raised disease activity and increased interleukin (IL-) 6. We asked to what extent IL-6 levels, the CRP polymorphism rs1205, and the type I interferon (IFN) gene signature affects the basal CRP levels in patients with SLE during a quiescent phase of the disease.

**Methods:**

CRP and IL-6 were analyzed in plasma from 57 patients meeting established classification criteria for SLE. The CRP polymorphism rs1205 was assessed and gene expression analyzed including four type I IFN-regulated genes (IGS).

**Results:**

CRP was increased in patients with detectable IL-6 levels (p=0.001) and decreased among IGS-positive subjects (p=0.033). A multiple linear regression model revealed IL-6 to have a positive association with CRP levels, whereas both IGS-positivity and CRP genotype (rs1205) AA/GA were negatively associated with CRP-levels.

**Conclusion:**

Our data offer an explanation to the modest CRP levels seen in viral infections and IFN-α driven autoimmunity and corroborate prior observations showing an IFN-α dependent downregulation of CRP. The latter observation, together with the fact that the CRP-lowering polymorphism rs1205 is overrepresented in human SLE, could explain low basal CRP and inadequate CRP-responses among patients with active SLE.

## Introduction

The acute-phase protein C-reactive protein (CRP) is a key actor in the clearance of bacteria and dying cells. Its pentameric structure encompasses an effector face with affinity for C1q and Fcγ-receptors and a recognition face with the ability to bind phosphocholine on e.g., dying cells and pathogens, but also nuclear constituents exposed during apoptosis (*i.e.*, snRNP and histones) (1, 2). These qualities enable CRP to contribute to efficient clearance of cell remnants and immune complexes by complement activation/modulation, opsonization, and phagocytosis, biological processes considered dysfunctional in systemic lupus erythematosus (SLE) (3, 4). Indeed, protective effects of CRP in the disease process have been demonstrated in animal models of lupus (5–7).

Due to increased CRP production from hepatocytes upon interleukin (IL-) 6 stimulation, CRP is widely used to monitor infectious and inflammatory conditions (8). With few exceptions, CRP reflects ongoing inflammation/tissue damage, seemingly without discrimination of the underlying trigger. However, whereas bacterial infections generally result in impressive CRP levels, the response in many viral infections is typically less pronounced (9). Similarly, modest CRP-responses are recorded in autoimmune diseases characterized by increased type I interferon (IFN) activity, e.g., SLE (8), and lack of correlation between IL-6 and CRP has been demonstrated in patients with SLE (10). Type I IFNs are strong activators of the anti-viral immune response, but may also contribute to autoantibody production in several autoimmune conditions (11).

IFN-α, the most studied type I IFN in autoimmunity, has previously been evaluated in relation to regulation of pentraxins (12–15). We have previously demonstrated an inhibitory effect of IFN-α in IL-6/IL-1β-induced CRP gene transcription and protein production from hepatocytes (12). Thus, the presence of IFN-α *per se* could contribute to modest CRP levels during IFN-α associated disease flares of type I IFN-driven autoimmunity as well as in viral infections. Furthermore, a combined effect of the CRP-lowering polymorphism of the CRP gene (rs1205) (16) and detectable IFN-α levels resulted in inability of CRP to reflect inflammatory activity among SLE patients (13). However, circulating IFN-α is difficult to detect with standard methods despite evidence of an increased gene transcription of type I IFN-regulated genes. Thus, a selection of type I IFN-regulated genes is typically assessed as a marker of an increased type I IFN activity, *i.e.*, the type I IFN gene signature (IGS).

The aim of this study was to confirm and extend the knowledge from previous *in vivo* and *in vitro* studies showing an impact of type I IFNs (12, 13, 15) and rs1205 (13) on CRP levels among patients with SLE. Herein, the objective was to evaluate the potential impact of the IGS and rs1205 in patients unbiased from high disease activity.

## Materials and Methods

### Subjects

All patients (**Table 1**) were followed within the frame of an observational research program at the University Hospital in Linköping (17). Each participant (*n*=57) was classified with SLE according to the 1982 American College of Rheumatology and/or the 2012 Systemic Lupus International Collaborating Clinics criteria (18, 19).

**Table 1:** Clinical characteristics of the 57 included patients with systemic lupus erythematosus (SLE).

Variable	Median (range) or frequency (%)
Age (years)	43 (23–63)
Females	50 (88)
Disease duration (years)	8 (1–35)
SLEDAI-2K	2 (0–8)
1982 ACR criteria fulfilled (number)	5 (3–9)
Ever (current or prior) tobacco smoker	14 (25)
*1982 ACR criteria fulfilled:*	
ACR 1: malar rash	23 (40)
ACR 2: discoid lupus	2 (3.5)
ACR 3: photosensitivity	26 (46)
ACR 4: oral ulcers	13 (23)
ACR 5: arthritis	45 (79)
ACR 6: serositis	22 (39)
ACR 7: renal disorder	19 (33)
ACR 8: neurologic disorder	6 (11)
ACR 9: hematologic disorder	38 (67)
ACR 10: immunologic disorder	35 (61)
ACR 11: antinuclear antibodies	57 (100)
*Ongoing immunomodulation:*	
Antimalarials	51 (90)
Prednisolone	30 (53)
Prednisolone dose (mg/day)	2.5 (0–15)
Mycophenolate mofetil	15 (26)
Methotrexate	5 (8.8)
Azathioprine	3 (5.3)
Other DMARDs/biologics	8 (14)
*^a^* *CRP genotype (rs1205)*	
Two major alleles (GG)	24 (44)
One minor allele (AG)	23 (42)
Two minor alleles (AA)	8 (15)

a Genotype analysis available for 55 patients.

ACR, American College of Rheumatology; DMARDs, disease-modifying anti-rheumatic drugs; SLEDAI-2K, SLE disease activity index 2000.

The patients donated peripheral blood and the disease activity, defined by SLE disease activity index 2000 (SLEDAI-2K), was recorded from their closest regular visit to rheumatologist. All patients were considered to have low disease activity but could be serologically active and clinically quiescent at sampling (20, 21). Each participant gave written informed consent, and the study protocol was approved by the regional ethics board in Linköping.

### Clinical Laboratory Analyses

Venous blood was drawn, and plasma was prepared and blood lipids were analyzed at the local Clinical Chemistry unit, including triglycerides, total cholesterol, high-density lipoprotein (HDL), low-density lipoprotein (LDL), non-HDL, IL-6, and CRP. We utilized a high sensitivity method for CRP with 0.15 mg/L as limit of quantification (LOQ). For IL-6, LOQ was 1.5 ng/L.

### Gene Expression

The type I IFN gene signature (IGS) was based on gene expression of IFI27, IFI44, IFI44L, and RSAD2 (22). Peripheral blood mononuclear cells (PBMCs) were isolated from heparinized whole blood by density gradient centrifugation and lysed by RLT-buffer. Total RNA was extracted using the RNeasy Mini Kit (Qiagen GmbH, Hilden, Germany). Quantification and purity of the RNA was assessed on a DS-11 spectrophotometer (DeNovix Inc. Wilmington, DE, USA). Total RNA was reverse transcribed using iScript™ cDNA synthesis kit (Bio Rad, Hercules, California, USA) to obtain cDNA for RT-qPCR. The RT-qPCR was performed by preheating for 10 min at 95°C, followed by 40 cycles of 95°C 15 s and 60°C for 60 s using StepOnePlus (Applied Biosystems, CA, USA). TaqMan™ Gene Expression assay (FAM) (Applied Biosystems, CA, USA) were used with the following primers from Thermo Fisher Scientific, IFI44L: Hs00199115_m1, RSAD2: Hs00369813_m1, IFI27: Hs00271467_m1, IFI44: Hs00197427_m1, GAPDH: Hs03929097_g1.

Fold‐change of gene expression was determined by the relative quantification method (ΔΔCt) after normalization to the housekeeping gene (GAPDH). A subsequent log-transformation was performed to achieve comparability between genes. IGS-score was expressed as mean of log-transformed fold change in relation to a control sample (consisting of a pooled sample from healthy control donors) of the four genes (detailed in **Supplementary Data 1**). The patient distribution revealed two groups regarding the IGS; a cut-off of 0.5 was applied to separate IGS-positive patients from IGS-negative.

### CRP Genotyping

DNA was extracted from peripheral blood using Qiagen Blood Midi Kit (Qiagen) and genotyping was performed using Illumina Infinium Global Screening Array-Multi Disease version3 (Illumina Inc. California, USA) at the SNP&SEQ Technology Platform at Uppsala University. The genotyping is detailed in **Supplementary Data 2**.

### Statistics

CRP levels were not normally distributed why Mann-Whitney U test, Kruskal-Wallis test, or Spearman’s correlation analysis were applied. CRP and IL-6 were log-transformed (10log) prior to linear regression analysis with CRP or IL-6 as dependent variables. A two-sided p-value of <0.05 was considered statistically significant.

## Results

### CRP Is Associated With IL-6 and IGS-Status

**Figure 1:**
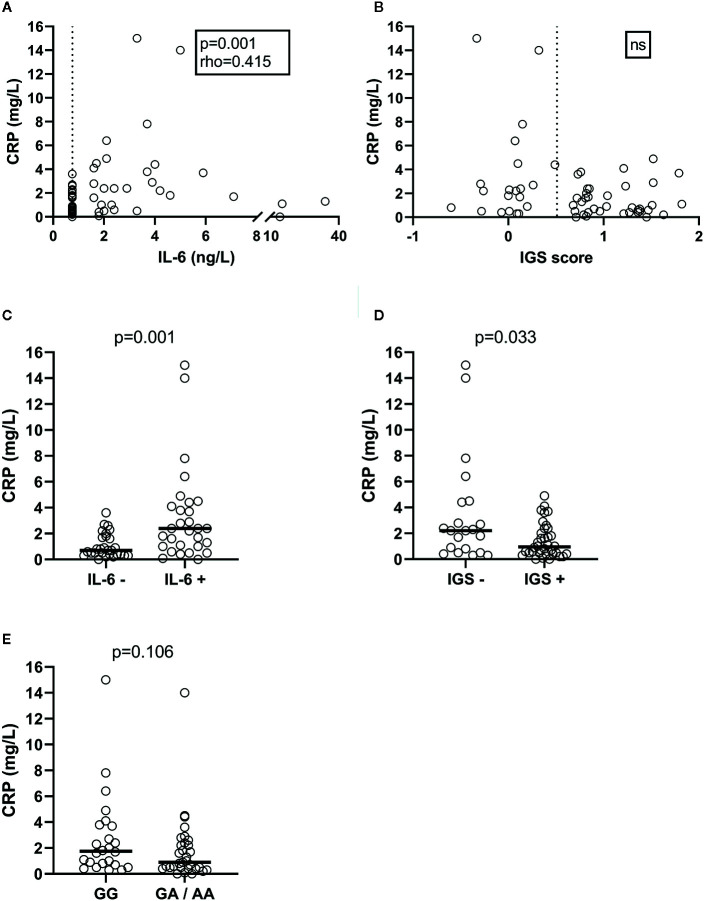
Plasma CRP levels in SLE patients, and the correlation and association with plasma IL-6 concentrations **(A)**, the IGS score measured in peripheral blood mononuclear cells **(B)**, detectable plasma IL-6 levels **(C)**, IGS positivity **(D)** and CRP rs1205 genotype **(E)**. Dashed lines represent cut-off levels for IL-6 detection and IGS positivity, respectively.

Levels of CRP correlated significantly with IL-6 (rho=0.415, p=0.001; **Figure 1A**) and with age (rho=0.292, p=0.027). No correlation was observed between CRP levels and IGS-score (**Figure 1B**). Since 28 patients had non-detectable IL-6, and 21 were judged IGS-negative, IL-6 and IGS were further tested as binary variables. CRP levels were significantly higher among those with detectable IL-6 (p=0.001; **Figure 1C**) and lower among IGS-positive subjects (p=0.033; **Figure 1D**). The CRP genotype (rs1205) was not significantly associated with CRP, neither based on number of rare alleles, nor based on presence or absence of one or two rare alleles (binary variables; **Figure 1E**).

No significant correlations between CRP and corticosteroid dose, SLEDAI-2K or blood lipids were observed. Neither did we observe any differences in CRP related to sex, tobacco smoking, or use of antimalarials, corticosteroids, mycophenolate mofetil, methotrexate or use of any immunosuppressive agent (disease-modifying anti-rheumatic drugs except antimalarials, *n*=28). Finally, CRP levels did not associate with disease phenotypes (*i.e.*, fulfilled classification criteria).

### IL-6, IGS, and CRP Genotype *Versus* CRP Levels

To evaluate impact of CRP genotype rs1205, IL-6, and IGS-positivity, these variables were included in a linear regression model with 10log CRP as the dependent variable. Age was further added as an independent variable due to its correlation with CRP.

**Figure 2:**
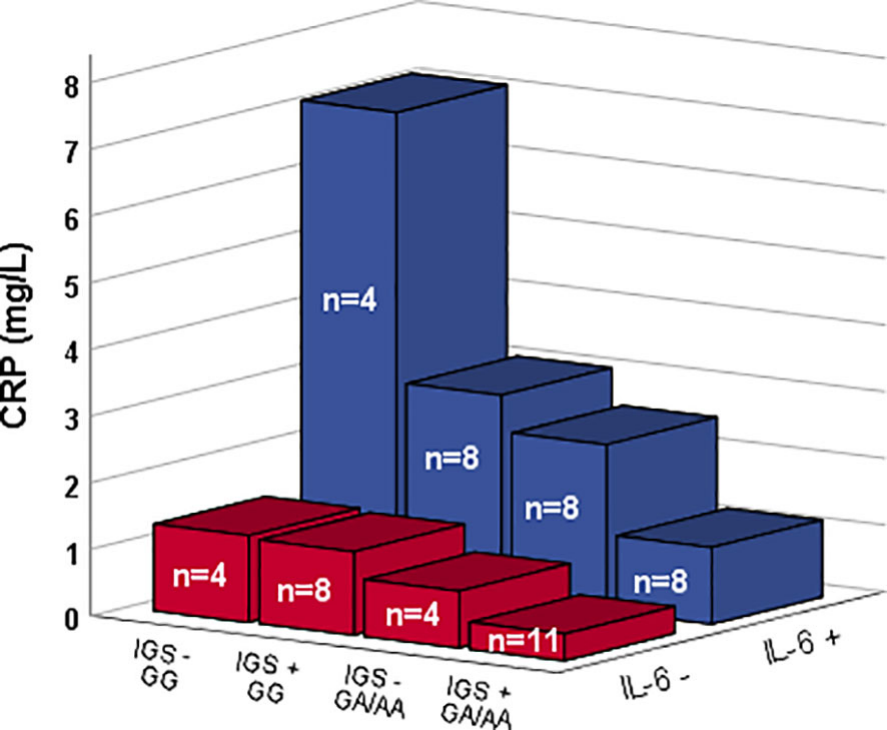
CRP levels among patients with and without detectable IL-6 levels (Z-axis) along with different CRP genotype (rs1205) and type I IFN gene signature (IGS) status (X-axis). Bars show median values.

A stepwise analysis revealed IGS-positivity (p=0.017) and alleles AA/AG of rs1205 (p=0.041) to be associated with low levels of CRP. Age and IL-6 levels did not associate with CRP in the regression analysis (**Table 2**). However, a regression analysis with IL-6 as binary variable resulted in a significant association of both IL-6 (p=0.012), CRP genotype (AA/AG) (p=0.030), and IGS-positivity (p=0.030) with CRP levels (**Table 2** and visualized in **Figure 2**).

**Table 2:** Variables associated with CRP levels (10log CRP) in stepwise linear regression analyses.

	p-value	Standardized beta	Model R^2^
**Model 1**			0.16
IGS positivity	0.017	−0.32	
rs1205 AA or AG	0.041	−0.27	
*Excluded in the model:*			
Age			
IL-6 levels			
**Model 2**			0.26
Detectable IL-6	0.012	0.32	
rs1205 AA or AG	0.030	−0.27	
IGS positivity	0.030	−0.27	
*Excluded in the model:*			
Age			

We observed no associations between IGS-status and CRP genotype, IL-6 (binary), or CRP genotype as determined by chi2 (Fisher’s exact test). A regression analysis with 10log IL-6 as the dependent variable and CRP genotype and IGS-status as dependent variables did not reach statistical significance, indicating that the effect of IGS and CRP genotype on CRP levels were independent of IL-6. 

## Discussion

The present study demonstrates that low CRP levels coincide with the CRP polymorphism rs1205 and the presence of an activated type I IFN system, as determined by the IGS-score. Our observations therefore add further support to an important role of type I IFNs in the regulation of circulating CRP *via* an inhibitory effect by IFN-α on CRP production (12, 15). Furthermore, we previously demonstrated an association between CRP and SLE disease activity when patients with the rs1205 minor allele and detectable serum IFN-α were excluded (13). Thus, it is plausible that our findings together with the fact that the CRP-lowering polymorphism rs1205 is overrepresented among SLE patients (16), explain the low basal CRP and inadequate CRP-responses in those with active SLE.

The extensive use of CRP as a biomarker motivate research aiming to unravel its regulation in different inflammatory conditions. Indeed, viral infections generally result in lower CRP-responses compared with bacterial infections (9), and SLE patients rarely mount a CRP-response that corresponds to their inflammatory activity (10, 23) or circulating IL-6 levels (10).

IL-6 is the main inducer of CRP-production, and it is therefore not surprising with a positive impact of IL-6 on circulating CRP (24). Herein, it was however evident that the CRP polymorphism, as well as type I IFN activity, coincides with a limited ability of IL-6 to induce a CRP-response. In fact, none of the IL-6-positive patients with the rs1205 minor allele and IGS-positivity reached a higher CRP level than 3.6 mg/L, *i.e.*, the highest CRP found among IL-6-negative subjects regardless of rs1205 genotype or IGS-status.

Hypothetically, other polymorphisms or presence of soluble receptors for *e.g.*, IL-6 and IFN-α could also impact CRP levels. As alternative explanations to the muted CRP response in SLE, increased consumption of CRP has been put forward. Increased complement consumption, due to inflammation and increased cell death, is a well-known feature of SLE flares (25). CRP has similar functions in the clearance of debris and could potentially be utilized and eliminated at a higher rate in patients with SLE. However, the elimination rate of CRP in SLE patients seems unaltered compared with healthy controls (26). Furthermore, anti-CRP autoantibodies, which are more frequently found in SLE, are not directed toward native circulating CRP, but toward epitopes that are exposed upon CRP dissociation on surfaces (27).

Limitations of the present study encompassed a relatively small number of patients and the lack of a replication cohort as well as a control group with unrelated inflammatory disease. The patients included had no known concomitant infections and low/stable disease activity, which reduce the potential impact of specific SLE-manifestations and comorbidities affecting the CRP-response. During bacterial infections, SLE patients usually present with an adequate CRP-response (28) which may be due to the massive increase of IL-6 that overrides the inhibitory effect of type I IFNs and/or genetic variants of CRP. In addition, bacterial infections could result in a shift of the immune activation with reduced type I IFN production. CRP itself has furthermore been shown to inhibit type I IFN production (29, 30) and it is tempting to speculate in a reciprocal regulation between pentraxins and type I IFNs. Finally, also leukocyte secretion/release of the closely related protein pentraxin-3 (PTX3) appear to be regulated by IFN-α *in vitro* (14).

Given the essential biological functions of pentraxins, such as CRP, in facilitating silent removal of cellular debris *via* Fc-receptors and by interacting with the complement system (1, 4) it is perhaps not surprising that administration of CRP to a murine lupus model prevent and reverse ongoing nephritis (5, 7). The crucial role of type I IFNs in SLE was once again demonstrated by the encouraging results of the phase 3 trial investigating the anti-IFN α/β receptor anifrolumab in patients with active SLE (31). These observations, together with our results, suggests that the consequences of CRP gene polymorphisms and IL-6/type I IFN interactions in SLE deserves further attention.

## Data Availability Statement

The original contributions presented in the study are included in the article/**Supplementary Material**. Further inquiries can be directed to the corresponding author.

## Ethics Statement

The studies involving human participants were reviewed and approved by the Regional Ethics Review Board in Linköping, Sweden. The patients/participants provided their written informed consent to participate in this study.

## Author Contributions

HE and CS conceived the original idea and project planning. HE, BG, JW, AAB, and CS contributed to the study design. CS collected clinical patient data. HE, BG, and M-LE carried out the laboratory work. M-LE, DL, and LR planned and supervised the genotyping. HE, BG, and CS analyzed the data. HE, LR, and CS drafted the manuscript. All authors contributed to the article and approved the submitted version.

## Funding

This study was funded by grants from the Swedish Research Council for Medicine and Health, the Swedish Society of Medicine and the Ingegerd Johansson donation, the Swedish Rheumatism Association, the Region Östergötland (ALF grants), the King Gustaf V’s 80-year Anniversary Foundation, the King Gustaf V and Queen Victoria’s Freemasons’ Foundation, the Gustafsson, Selander, Alfred Österlund, Anna-Greta Crafoord and the Greta and Johan Kock’s Foundations.

## Conflict of Interest

The authors declare that the research was conducted in the absence of any commercial or financial relationships that could be construed as a potential conflict of interest.
